# Primary and Secondary Negative Symptoms in Schizophrenia

**DOI:** 10.3389/fpsyt.2021.766692

**Published:** 2022-01-03

**Authors:** Sergey N. Mosolov, Polina A. Yaltonskaya

**Affiliations:** ^1^Moscow Research Institute of Psychiatry, Moscow, Russia; ^2^Russian Medical Academy of Continuous Professional Education, Moscow, Russia

**Keywords:** schizophrenia, course trajectory, secondary negative symptoms, primary negative symptoms, depression, extrapyramidal symptoms

## Abstract

The negative symptoms of schizophrenia include volitional (motivational) impairment manifesting as avolition, anhedonia, social withdrawal, and emotional disorders such as alogia and affective flattening. Negative symptoms worsen patients' quality of life and functioning. From the diagnostic point of view, it is important to differentiate between primary negative symptoms, which are regarded as an integral dimension of schizophrenia, and secondary negative symptoms occurring as a result of positive symptoms, comorbid depression, side effects of antipsychotics, substance abuse, or social isolation. If secondary negative symptoms overlap with primary negative symptoms, it can create a false clinical impression of worsening deficit symptoms and disease progression, which leads to the choice of incorrect therapeutic strategy with excessive dopamine blocker loading. Different longitudinal trajectories of primary and secondary negative symptoms in different schizophrenia stages are proposed as an important additional discriminating factor. This review and position paper focuses primarily on clinical aspects of negative symptoms in schizophrenia, their definition, phenomenology, factor structure, and classification. It covers the historical and modern concepts of the paradigm of positive and negative symptoms in schizophrenia, as well as a detailed comparison of the assessment tools and psychometric tests used for the evaluation of negative symptoms.

## Introduction

Negative symptoms are a core component of the schizophrenia syndrome. Negative symptoms can be primary symptoms, which are intrinsic to the underlying pathophysiology of schizophrenia, or secondary symptoms that are related to psychiatric or medical comorbidities, adverse effects of treatment, or environmental factors. Although negative symptoms are diverse and difficult to differentiate, careful assessment, timely identification, and provision of adequate therapy are required. More than half of patients with chronic schizophrenia exhibit at least one negative symptom (1), and the prevalence of persistent negative symptoms following the first psychotic episode is reaching 11–37% (2). In a multicenter retrospective study (*n* = 1,452), the majority of the patients (57.6%) diagnosed with schizophrenia spectrum disorders had at least one or more negative symptoms, while primary negative symptoms were reported in 12.9% of the patients; in another study (*n* = 7,678), 41% of the patients had at least two negative symptoms (3). In a 15-year prospective study in patients with schizophrenia and schizoaffective and affective disorders, the prevalence of negative symptoms was found to be high: 75, 68, and 44%, respectively (4). In the course of the disease, negative symptoms occur very early, often in ultrahigh risk state, and this very likely predicts the transition to schizophrenia (5). In a retrospective study of the onset of schizophrenia in 4,707 patients seeking psychiatric assistance, negative symptoms were observed in 95% of the examined subjects. Furthermore, at the pre-manifest stage of the disease, 32.7% of the subjects demonstrated social withdrawal with increasing self-isolation, 25.8% developed asthenoneurotic and asthenodepressive symptoms, and only 7% showed apatho-abulic manifestations (6). Apart from decreasing patients' quality of life, negative symptoms are associated with impaired daily life functioning, social relationships, and the professional activity of such patients (7–9), as well as with rarer achievement and poorer quality of remissions in the course of the disease (10, 11). As compared to positive symptoms, negative symptoms show no tendency toward spontaneous improvement in the course of the disease and respond poorly to treatment with currently used antipsychotics (12–14).

## Historical Evolution of The Concept of Negative Symptoms In Schizophrenia

The first theories regarding negative symptoms in schizophrenia date back to the beginning of the 19th century, when J. Haslam, a British physician, described a mental disease in young people that was characterized by a long-lasting “depressed sensitivity and emotional indifference” (15). However, the first conceptual justification for differences between positive and negative symptoms was given by the British neurologist J. R. Reynolds, who proposed to distinguish plus-symptoms associated with distortion or superfluity of natural functions (delusions, hallucinations, convulsions, pathological movements, etc.) and minus-symptoms associated with loss or deficit of natural functions or misbehavior. This referred first of all to motivation and interest, although it was indicated that any function could be lost, e.g., memory, sensitivity, motor activity, etc. (16). That said, negative and positive symptoms were not mutually exclusive and could coexist. Another British neurologist J. H. Jackson considered negative symptoms as a stable impairment of higher cortical functions, whereas periodic positive symptoms were regarded as a phenomenon of excessive functioning (17). He considered the relationship between positive and negative symptoms within the framework of the evolutionary theory of stratification applied to mental disorders (“dissolution of the nervous system”) and believed that loosening of control of higher cortical functions (negative symptoms) leads to disinhibition of the activity of phylogenetically more ancient and primitive lower subcortical structures. This manifests as a pathological response (positive symptoms, primarily affective and psychotic symptoms) that is effectively the secondary compensatory phenomenon.

The German psychiatrist Emil Kraepelin was the first to point out the significance of negative symptoms (restricted emotional expression and avolition, cognitive impairment, and social withdrawal) in patients with dementia praecox and to set them against productive symptoms, such as hallucinatory-delusional and catatonic-hebephrenic symptoms (18). Furthermore, if the latter was considered as reversible (relapsing), then negative symptoms were regarded as irreversible, progressive, and leading to dementia, i.e., as a residual deficit or “defect.”

Unlike Kraepelin, who, despite the acknowledgment of a simple form of schizophrenia later on, still did not consider negative symptoms obligatory for dementia praecox, E. Bleuler, who introduced the term “schizophrenia” meaning “schisis” or “splitting of antagonistic functions” in 1911, immediately tried to define its basic manifestations with the emphasis on negative symptoms. According to Bleuler, negative symptoms comprised weakening of the association process, inadequacy or affective flattening, and volitional disorders, including ambivalence and autism (19). The irregular development and known reversibility of the main (negative) and primary (somatic) symptoms were assumed. Interestingly, speech and written language disorders, memory impairment, and personality changes were also referred by the author to secondary accessory symptoms, along with affective, catatonic, and psychotic symptoms. Bleuler was mostly interested in psychological and even psychodynamic mechanisms of schizophrenia development, viewing those as losing associations, rather than in the course and prognosis of the disease. This understanding, relying on the detection of obligatory (basic) negative symptoms, led to a significant expansion of the diagnostic spectrum of schizophrenia to include early, mild, and latent forms of the disease with insignificant intensity or even complete absence of any given positive psychopathological symptoms.

In the 1970s, the German psychiatrist J. Strauss asserted the primary and chronic nature of negative symptoms, while considering positive symptoms as a non-specific transient reaction to stress (20). Another of his compatriots, G. Huber, and his followers developed the concept of “basic symptoms” in schizophrenia, by which they meant primary subjective experiences of patients directly related to a pathological process in the brain and forming the basis for the development of complex secondary symptoms (21, 22). In this context, basic symptoms were regarded as deficit symptoms, which are subjectively evaluated by patients as insufficiency or defect at the so-called basic stages of the disease, namely, in the pre-psychotic (prodromal), reversible post-psychotic, or irreversible state of “pure” defect. In turn, productive psychotic symptoms were deemed as psychoreactive, adaptive, and personality-mediated “epiphenomena of schizophrenia.” All basic stages can be reversed, and, therefore, the progression of the disease does not lead to the inevitable formation of a defect. Only a non-specific reduction in the overall level of mental energy is irreversible (the so-called pure asthenic defect) if it lasts for more than 2 years (21).

A 20-year follow-up study of patients with schizophrenia has shown that persistent basic symptoms occurred more frequently than characteristic personality changes. This stance of focusing on non-specific asthenic and pseudo-organic (somatic) deficit disorders and leveling the significance of peculiar (specific) personal, cognitive, and emotional disorders in schizophrenia significantly distinguishes the concept of G. Huber from the dominant views in Russian psychiatry. Following the ideas of Janzarik (23), the author uses a taxonomic multilevel model of basic symptoms and identifies the following: (1) “substrate-active” disorders with hyperfunction of dopaminergic structures, when negative symptoms are the consequence of active functional inhibition (e.g., inability to concentrate, deautomatization of motor actions, apatho-adynamic disorders, and poor speech production); (2) “substrate-negative” disorders accompanied by exhaustion and hypofunction of dopaminergic structures, when a persistent torpid to traditional antipsychotic therapy reduction of affective and energy levels is developed; (3) “substrate-deficient” disorders associated with final structural changes in the brain and loss of functions (e.g., persistent asthenic symptoms—“pure defect”) (24).

**Figure 1:**
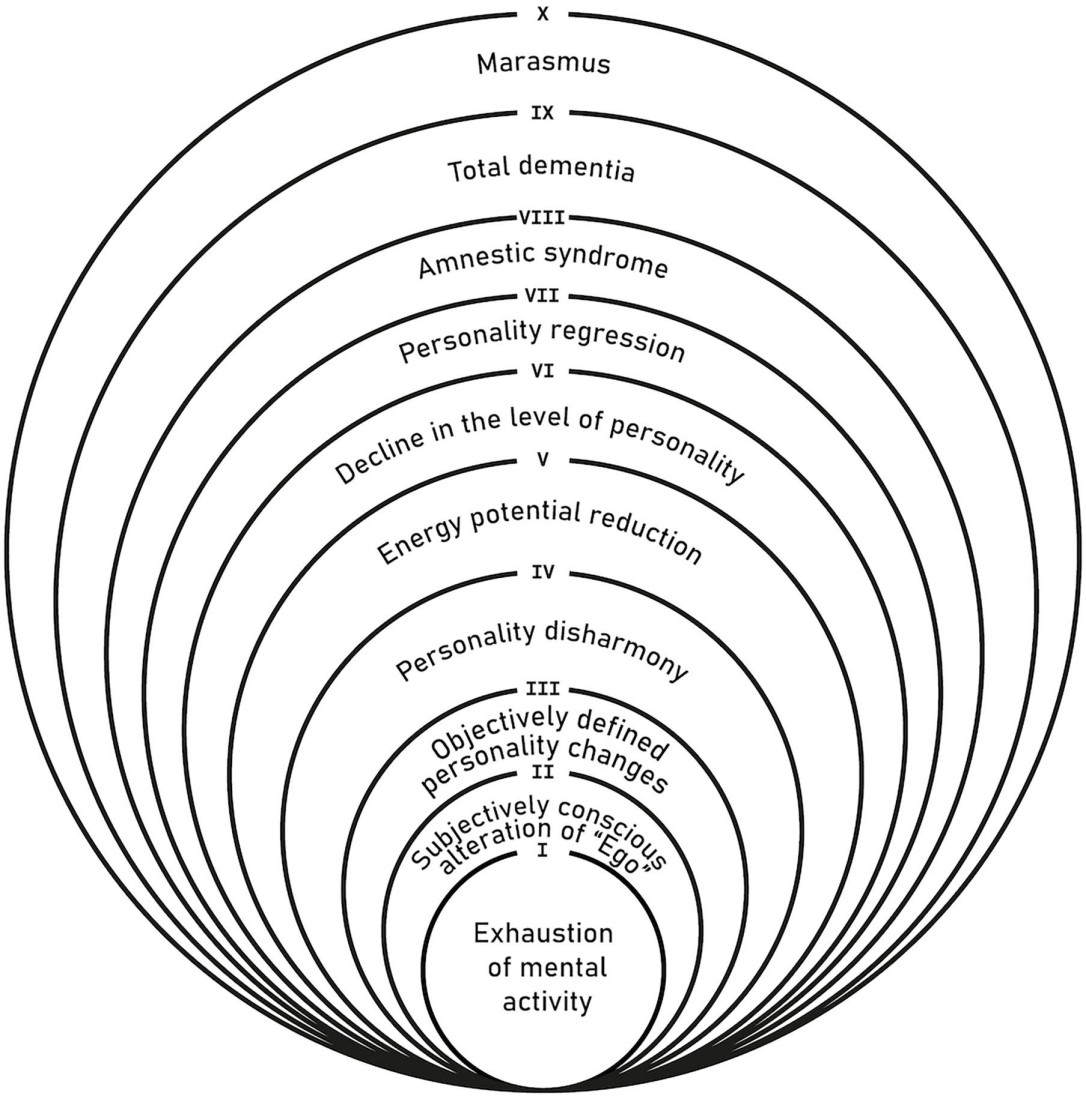
Hierarchy of negative symptoms by the level of their severity (25).

An important role in the development of the concept of negative (deficit) symptoms in schizophrenia was played by the Russian school of psychiatry headed by A. V. Snezhnevsky, who paid a lot of attention to specific autistic personality changes and described an entire range (continuum) of deficiency symptoms—from hardly noticeable for the patient's immediate circle, social withdrawal with the prevalence of frailty and excessive vulnerability, to a pronounced decrease in energy potential, lack of initiative, and emotional impoverishment to the extent of outright apatho-abulic dementia with regression of the earliest acquired automatic daily activity skills (25). Negative or deficit symptoms in schizophrenia include many reversible or persistent impairments—from asthenization of mental activity to pronounced state of mental marasmus, including personality changes, amnestic syndrome, and dementia, which could be ranked according to their severity (Figure 1). Each circle of a higher level includes all the underlying and less specific syndromes—from mental exhaustion and reduced energetic potential to personality regression and total dementia. For a better understanding of the terminology of this model, we added a small glossary of terms in the addendum (Appendix 1). Following J. H. Jackson, A. V. Snezhnevsky and most Russian psychiatrists interpret negative symptoms as a loss or reduction of mental function (minus-symptoms), i.e., loss of any mental ability due to the damage of the central nervous system. Since the deficit of mental functions is understood as irreversible damage caused by the disease, so-called “scar” by Kraepelin, negative symptoms are often associated with a notion of the deficit or “defect.” This negative or deficit syndrome is considered nosospecific for schizophrenia (26). The most common deficit symptoms are falling intellectual activity, autism or social withdrawal, willful decline or avolition, impoverishment of emotional reactions or blunted affect, reduction of mental activity, or apathy. Personality changes (“personality shift”) and “specific” thought disturbances (loosening of associations, derailments, tangential thinking, etc.) are also included in the definition of “schizophrenic defect.” The originality of Snezhnevsky's clinical approach primarily lied in the dynamic analysis of the disease course, within which progression of negative symptoms was considered in close relation to syndrome kinetic pattern of different psychotic symptoms. For A. B. Smulevich, the pupil and the most consistent follower of Snezhnevsky, the concept of “schizophrenic defect” also includes persistent positive residual symptoms and some personality (pseudopsychopathic) deviations like “Verschrobener” or bizarre/eccentric behavior with dysbulia (weakness and uncertainty of volition) without necessarily premorbid evidence of personality disorder (27). The intertwining and dynamic of all these residual symptoms are very important for determining a more accurate definition of remission in schizophrenia and for the prognosis of long-term therapeutic effect (28). We believe that the severity, frequency, and presentation of various negative symptoms are different in specific forms and stages of schizophrenia (29).

In the 1980s, the British researcher T. Crow formulated a dichotomous hypothesis of schizophrenia highlighting two independent pathological processes: (1) type I schizophrenia with a predominance of negative symptoms and (2) type II schizophrenia with a predominance of positive symptoms. Positive schizophrenia was characterized by a nearly normal premorbid period, relatively acute onset of the disease, prevalence of psychotic symptoms, clear episodic course (exacerbations and attenuation of symptoms), relatively good social and working adaptation, nearly normal performance on cognitive tests, and absence of structural changes in the brain evaluated using different scanning techniques. Negative schizophrenia was characterized by the presence of cognitive and negative symptoms in the premorbid period, low level of education, gradual or latent onset of the disease, predominant negative symptoms (emotional blunting, poor speech, anhedonia, attention deficit, lack of motivation, and volitional impulses), chronic or malignant course, social and working disadaptation, poor performance on cognitive tests, and different structural changes in the brain including signs of cerebral atrophy. The subtypes also differed in their response to dopamine-blocking antipsychotic therapy. In negative schizophrenia, unlike positive one, low response to antipsychotics was observed. Post-mortem and neuroimaging studies have revealed that in positive schizophrenia, different cerebral structures demonstrate predominant dopaminergic activity and increased density (hypersensitivity) of D_2_-receptors, while negative schizophrenia is characterized by hypodopaminergic activity (mostly in the pre-frontal cortex), normal or reduced density of D_2_-receptors, reduced glucose metabolism, neuronal loss, and their decreased functional activity (reduced gray matter volume and number of spikes in the frontal cortex). In a factor analysis of psychopathological symptoms, T. Crow had found no relationship between positive and negative symptoms; however, negative symptoms correlated with cognitive impairment, low social functioning, and residual neurological symptoms (30).

## Position of American Researchers

At the end of the 1970's, American researchers criticized the existing criteria for diagnosis of schizophrenia based on the first-rank symptoms of K. Schneider and associative cognitive dysfunction of E. Bleuler due to their lack of specificity (20, 31–33). Based on the old concept of J. R. Reynolds and J. H. Jackson, they proposed to allocate two main pathological syndromes of schizophrenia, i.e., positive and negative syndromes. A particular credit for describing and quantifying the symptoms comprising these syndromes goes to the psychologist from Iowa, Nancy Andreasen, who had developed special scales for psychometric assessment of positive (SAPS) and negative (SANS) symptoms. The SANS scale includes the following five domains of negative symptoms:

1) affective flattening or blunting, unchanging facial expression, amimia, decreased spontaneous movements, paucity of expressive gestures, poor eye contact, affective non-responsivity, inappropriate affect, lack of vocal inflections (monotone voice);
2) alogia (poverty of speech): reduction in the quantity of speech, poverty of content of speech, blocking, breaks in thought, increased latency of response;
3) avolition—apathy: poor grooming and hygiene (including personal hygiene), impersistence at work or school, physical anergia;
4) anhedonia—asociality: reduced interest in recreational activities, reduced sexual interest and activity, inability to feel intimacy and closeness, difficulties with relationships with friends and peers;
5) attention impairment: social inattentiveness, inattentiveness during mental status testing.

Eventually, N. Andreasen concluded that there were two fundamentally different forms of schizophrenia, with the prevalence of positive or negative symptoms. However, unlike T. Crow, she admitted the existence of a continuum of intermediate forms with mixed symptoms (33). Patients with predominant negative symptoms were characterized by a lower level of premorbid adaptation and social functioning, more pronounced cognitive impairment, and signs of cerebral atrophy.

Factor analysis of symptoms using the SANS scale revealed three main factors: (1) affective flattening (reduced expressiveness); (2) disturbed attention—alogia (poverty of speech); (3) reduced social motivation (abulia, apathy, anhedonia, asociality). In different studies, the most frequently observed symptom was reduced social motivation; somewhat rarely seen are symptoms of decreased emotional expressiveness (34–36).

These and other new studies conducted over the last decade have shown the necessity to exclude negative symptoms that are not directly associated with emotional and motivational deficits (e.g., attention disturbance, poverty of content of speech, increased latency of response, inappropriate affect), as well as those overlapping the other dimensions of schizophrenia, such as cognitive disorganization, cognitive impairment, and depressive symptoms. The consensus had been reached regarding the inclusion of the following five major factors in the concept of negative symptoms (37):

1) anhedonia—inability to feel pleasure;
2) avolition (apathy)—lack of energy and initiative, loss of interest for usual activity;
3) social withdrawal—disturbed social activity and avoidance of interpersonal contacts;
4) alogia—negative cognitive disorder, narrowing of speech range, and poverty of content of speech;
5) emotional (affective) flattening or blunting, reduced emotional response to stimuli.

This five-factor model of negative symptoms in schizophrenia has recently been also confirmed with independent network analysis (38).

Volitional impairment and, above all, goal-oriented behavior seem likely to be core negative symptoms in schizophrenia, as in this disease, the reward system, and goal-directing planning are disturbed (39). Patients can experience pleasure from a particular moment in the present but do not extrapolate it to the future, suffering from so-called anhedonia paradox (40). Avolition, abulia, apathy, anhedonia, and social withdrawal apparently have a single underlying mechanism based on disturbances in motivational sphere, including a decrease in motivation for social activity. The other component is associated with impairment of emotional expression and includes poverty of speech (alogia) with a paucity of spontaneous speech and affective flattening (decreased facial expressiveness, voice intonations, and gesticulation).

A two-factor structure of negative symptoms (motivational–volitional and emotional–expressive disorders) has been confirmed in several studies (37, 41, 42). Besides, such division is also confirmed by the trajectory of their development in the course of the disease, including their long-term stability and relationship with functional outcomes (10). It cannot be ruled out that they have different neurophysiological and neurochemical mechanisms and different responses to drug therapy (43). However, recent cohort studies have again shown a number of advantages and validity of a five-factor model of negative symptoms in schizophrenia (44, 45).

Another clinical approach to studying schizophrenia in patients with predominant negative symptoms was proposed by W. Carpenter, who designated schizophrenia with deficit syndrome as a particular subtype of the disease called “deficit schizophrenia” (46, 47). The authors proposed the following diagnostic criteria for a given subtype:

1) presence of at least two of the following negative symptoms is required:
  - restricted affect;
  - diminished emotional range and reduced variability of emotional reactions;
  - poverty of speech;
  - curbing of interests;
  - diminished sense of purpose;
  - diminished social drive;
2) symptoms have been present for the preceding 12 months and during periods of clinical stability;
3) symptoms are of primary nature with regard to the disease.

The deficit syndrome occurs in approximately 15% of patients experiencing the first episode of schizophrenia, in 20–25% of inpatients, and in 15–20% of all cases of schizophrenia (48). It is also found throughout the long-term follow-up period and remains stable in the course of the disease (49, 50). In contrast to all other variants of the disease, patients with deficit schizophrenia consistently demonstrate the worst therapeutic and social prognosis (51). For instance, comparison of the efficacy of haloperidol and clozapine in a small randomized clinical trial (RCT) did not reveal any differences in the effect of drug therapies on negative symptoms, although clozapine reduced positive symptoms to a greater extent (52). Notwithstanding several studies that emphasized the clinical uniqueness of deficit schizophrenia (53), its identification as a separate form can be hampered primarily because of practical difficulties of differentiating primary and secondary negative symptoms.

In the RCTs evaluating the effect of new treatments on negative symptoms in order to faster identify a homogeneous group of patients with predominant negative symptoms, the more pragmatic concepts of dominant and persistent negative symptoms have gained widespread use.

Persistent negative symptoms (PNSs) include pronounced negative symptoms that persist for at least 6 months with no and/or minimal depressive symptoms and pseudoparkinsonism; they persist during the period of clinical stability (remission) on the background of low intensity or absence of positive symptoms and interfere with everyday functioning and social activities (54). The diagnosis of PNS rests on three criteria: (1) presence of a clinically stable negative syndrome for at least 3 months before psychiatric evaluation; (2) negative symptoms positive and negative syndrome scale (PANSS) score >24; (3) mild intensity (<4 points) of such symptoms as psychomotor agitation, hyperactivity, hostility, suspiciousness, negativism, and deficits in control of motivation as evaluated by PANSS. PNSs present a broader concept as compared to deficit syndrome. Although there is no clear distinction between primary and secondary negative symptoms, the criteria for PNS have been used in several RCTs (1, 55, 56). The prevalence of PNS depending on the severity of positive symptoms and duration of follow-up is 3.8–31.5% (54). In 23.7% of patients with first episode of schizophrenia, stable PNSs have been observed for 3 years (57). PNSs are more common in men, unemployed, subjects with a longer period of untreated psychosis, lack of insight, and a more significant, as compared to other forms of schizophrenia, decrease in the premorbid progress in studies and in social and cognitive functioning (2). However, as compared to patients with deficit schizophrenia, subjects with PNSs demonstrate, as a rule, less pronounced premorbid deficit and cognitive impairment. The concept of PNSs is now widely recommended both in practice and in clinical studies because it takes into account the parameter of symptom stability and allows to differentiate primary and secondary negative symptoms (58).

Notwithstanding the necessity of reaching a consensus on the definition of negative symptoms in RCTs, the inclusion criteria for patients with negative symptoms differ significantly (37, 54). Apart from PNSs, there are also dominant or predominant negative symptoms (DNSs). The latter is usually seen in patients with a negative PANSS composite index. However, in RCTs, these criteria are often modified, and additional conditions are added, e.g., intensity of three negative symptoms of not <4 points or of two negative symptoms of not <5 points amid low (below 4 points) intensity of two or more positive symptoms (59, 60). According to Riedel et al. (61), the DNS criteria include the following: (1) mandatory presence of three negative symptoms of moderate severity (at least 4 points) or two severe negative symptoms (at least 5 points); (2) PANSS total negative subscale score of at least 6 points higher than that on the positive symptom subscale (negative composite index); (3) negative symptom PANSS total score is equal to or >21 (61); (4) severity of the symptoms described in items 1 and 2 is determined by the total score on the PANSS positive symptom subscale and should be not more than 19 points (62).

Differences in the definition of DNS are in part associated with disagreement among regulatory agencies on their use in RCTs. The US Food and Drug Administration insists that negative symptoms recognized as pronounced should be used only for patients with high severity of negative symptoms, whereas the European Medicines Agency has introduced an additional criterion of “no-to-little positive symptoms,” which brings this group of negative symptoms closer to PNS (63). In the large-scale CATIE trial that enrolled 1,447 patients with schizophrenia, two-thirds of the patients had clinically significant negative symptoms, and in 18.9% of them, these symptoms were predominant (64).

## Diagnosis and Psychometric Assessment of Negative Symptoms

The clinical diagnosis of negative symptoms is a rather difficult task. It is much easier to detect and evaluate positive symptoms owing to their intensity and direct response to antipsychotic therapy. The diagnosis of negative symptoms requires objective information and careful observation of the patient's behavior including their ability to express emotions, motivation in different spheres of personal and social activity, and interest in receiving treatment. Special scales for differential diagnosis of negative symptoms have been developed, including SANS (33) and PANSS (65). Although the PANSS includes a subscale consisting of seven negative symptoms, a subsequent factor analysis with independent evaluation has shown that four symptoms from the general psychopathological subscale are also related to the PANSS (mannerism and posturing, motor retardation, disturbance of volition, and active social withdrawal). This new cluster consisting of 11 symptoms forms the so-called Marder factor (66), which has recently been used mostly in the assessment of the negative dimension in schizophrenia using the PANSS scale. Interestingly, in the CATIE trial conducted in 1,447 schizophrenic patients to comparatively evaluate the efficacy of atypical antipsychotics without the involvement of pharmaceutical companies, the Marder factor appeared to be the strongest predictor of global patient functioning as compared to any other PANSS factors or symptoms both at baseline and after 18 months of any therapy. New scales and structured interviews for evaluation of negative symptoms include the Negative Symptom Assessment Scale (NSA-16 and NSA-4) (67, 68), Brief Negative Symptom Scale (BNSS) (69), and Clinical Assessment Interview for Negative Symptoms (CAINS) (42). The last two scales contain 13 items, take 15–30 min for evaluation, and allow to differentiate between the emotional and motivational negative symptom clusters.

In addition to physician-rated scales, there are questionnaires for Self-evaluation of Negative Symptoms (SNS) and Motivation and Pleasure Scale–Self-Report (MAP-SR), which seem to be promising tools for routine clinical screening (70, 71). An important aid in diagnosis, particularly in complex cases that require the differentiation between depressive and negative symptoms, special psychometric scales such as the Calgary Depression Scale for Schizophrenia (CDSS) enable the evaluation of the severity of depression in schizophrenic patients (72). The Maryland Trait and State Depression scale (MTSD) enables the identification of depressive symptoms in the clinical picture of schizophrenia (73). The CDSS seems to be a more accurate tool for differential diagnosis; the uniqueness of CDSS compared to HAMD is that CDSS factors are stable over the course of the disease and appear independent of positive and negative symptoms.

A detailed analysis of the advantages and disadvantages of the various scales for assessing negative symptoms was recently provided by Lincoln et al. (74). In addition to clinical observation and structured interviews used for detection of negative symptoms, new registration methods, such as actigraphy or examination using a smartphone, provide an opportunity to detect symptoms and increase the level of activity of patients in their natural environment (75).

## Differential Diagnosis of Primary and Secondary Negative Symptoms

The distinction between primary and secondary negative symptoms has important diagnostic and therapeutic significance. Primary negative symptoms are an integral part of the phenomenon of schizophrenia and are characterized by a longer manifestation throughout the disease (13). Secondary negative symptoms may result from positive symptoms (e.g., social withdrawal based on suspicion in persecutory delusions), comorbid depression, side effects of antipsychotics, side effect of antidepressants, or use of psychoactive substances (e.g., cannabis) or can be caused by social deprivation resulting from long-term hospitalizations and loss of close relatives.

Some neuropsychological disorders, especially depression and parkinsonism, have phenomenological similarities with negative symptoms in schizophrenia (Table 1). Therefore, in clinical practice, it can be very difficult to distinguish emotional blunting from depressive anhedonia, anesthesia, apathy, and mental indifference or amimia in parkinsonism. Apatho-abulic disorders can be easily mixed up with depressive motor retardation and parkinsonian akinesia, and cognitive impairment, and disturbance of associative thinking—with depressive retardation often accompanied by difficulties with concentration or bradypsychia, cognitive dysfunction, and impaired speech production in parkinsonism. Schizophrenic autism may be difficult to differentiate from social withdrawal in depression or forced restriction of social contacts in parkinsonism. Many foreign and Russian authors have paid attention to difficulties in diagnosing such disorders (76–78).

**Table 1:** Differential diagnosis of negative symptoms.

**Negative symptoms**	**Depression**	**Parkinsonism**
Emotional blunting	Anhedonia, indifference, anesthesia	Indifference, amimia
Apatho-abulic disorders (reduced psychic energy potential)	Motor retardation	Akinesia, increased muscle tone
Cognitive impairment, poverty of speech and associative thinking	Mental retardation, difficulties with concentration	Bradypsychia, cognitive impairment, decreased vigilance, difficulties with concentration, impaired speech production
Autism	Social withdrawal	Forced restriction of social contacts

The following differential diagnostic considerations can be suggested for secondary negative symptoms, even though a definitive distinction is often impossible.

1. Common Psychopathological Aspects of Negative Symptoms and Depression Include Anhedonia, Apathy, Suppression of Affective Sphere, Social Isolation, and Asthenia. Depressed Mood and Sleep Disorders Are More Often Observed During Depression. Besides, in Depression, the Leading Symptoms Include low Self-Esteem, Feelings of Despair, and Ideas of Guilt. Autonomic Symptoms, Circadian Rhythm Disruption, and Suicidal Ideation Are Also Common (79). In a special study, it has been found that the most important differentiating features for depression in schizophrenia patients are low mood and pessimistic and suicidal ideation and for negative syndrome are alogia, flattened affect, and social withdrawal (80).
2. Positive symptoms are often accompanied by daily life restrictions, for instance, delusions of persecution or hallucinations can lead to social isolation and anhedonia. Such negative symptoms become less intense as the hallucinations and delusions are reduced with antipsychotic therapy (81).
3. The other cause of secondary negative symptoms can be the inadequate use of antipsychotic therapy. Excessive sedation or extrapyramidal symptoms (EPSs) can lead to flattening of affect and motor impairments like stiffness and reduced activity (82). In such cases, differential diagnosis is made by establishing links between the onset of symptoms and the start of antipsychotic therapy or the addition of a new antipsychotic with strong dopamine-blocking properties.
4. Chronic substance abuse, e.g., cannabis, is associated with the so-called amotivational syndrome, which can clinically overlap with the existing negative symptoms imitating the latter (83). In such cases, differential diagnosis is made by collecting a detailed history of substance use, performing laboratory tests for their presence in the blood or urine, and following up symptoms during the abstinence period.
5. Less frequently, the development of secondary negative symptoms is related to environmental conditions, for instance, social deprivation during long-term hospitalization (81, 82). In this case, the issue of differential diagnosis is solved through obtaining detailed anamnestic and follow-up data of symptoms when a patient changes his/her type of activity or place of residence.

**Figure 2:**
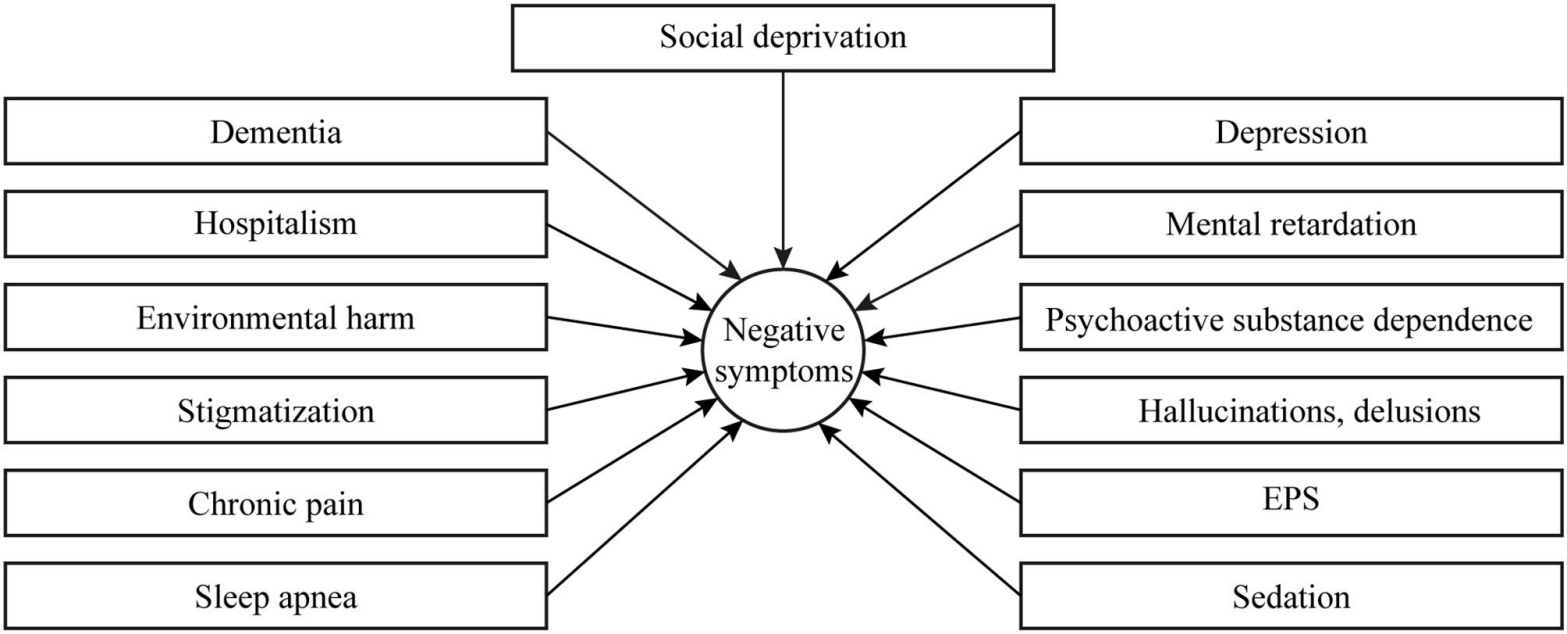
Secondary negative symptoms in schizophrenia. EPS, extrapyramidal symptom.

The above listed secondary negative symptoms are seen most frequently in clinical practice; however, their possible variants are much more diverse and have a broader spectrum of causes (neurological, somatic, social, or environmental) (Figure 2). Nonetheless, their careful detection and identification of their causes are of enormous practical importance, as it determines further therapeutic strategy. Unfortunately, in Russia, patients with severe stable negative symptoms rarely seek help from a psychiatrist, and they are usually pushing back against the idea of treatment. At the same time, physicians are seldom able to distinguish between primary and secondary negative symptoms and do not always attempt to treat negative symptoms because they consider them irreversible. To a certain degree, such therapeutic negativism in Russia is related to the prevalence of the “defect” concept of E. Kraepelin and A. V. Snezhnevsky. In a recent online survey of 807 members of the Russian Soci ety of Psychiatrists, only 51% of physicians specifically inquired about the presence of negative symptoms for diagnostic purposes, and 58% of those analyzed whether these symptoms were primary or secondary (84).

**Figure 3:**
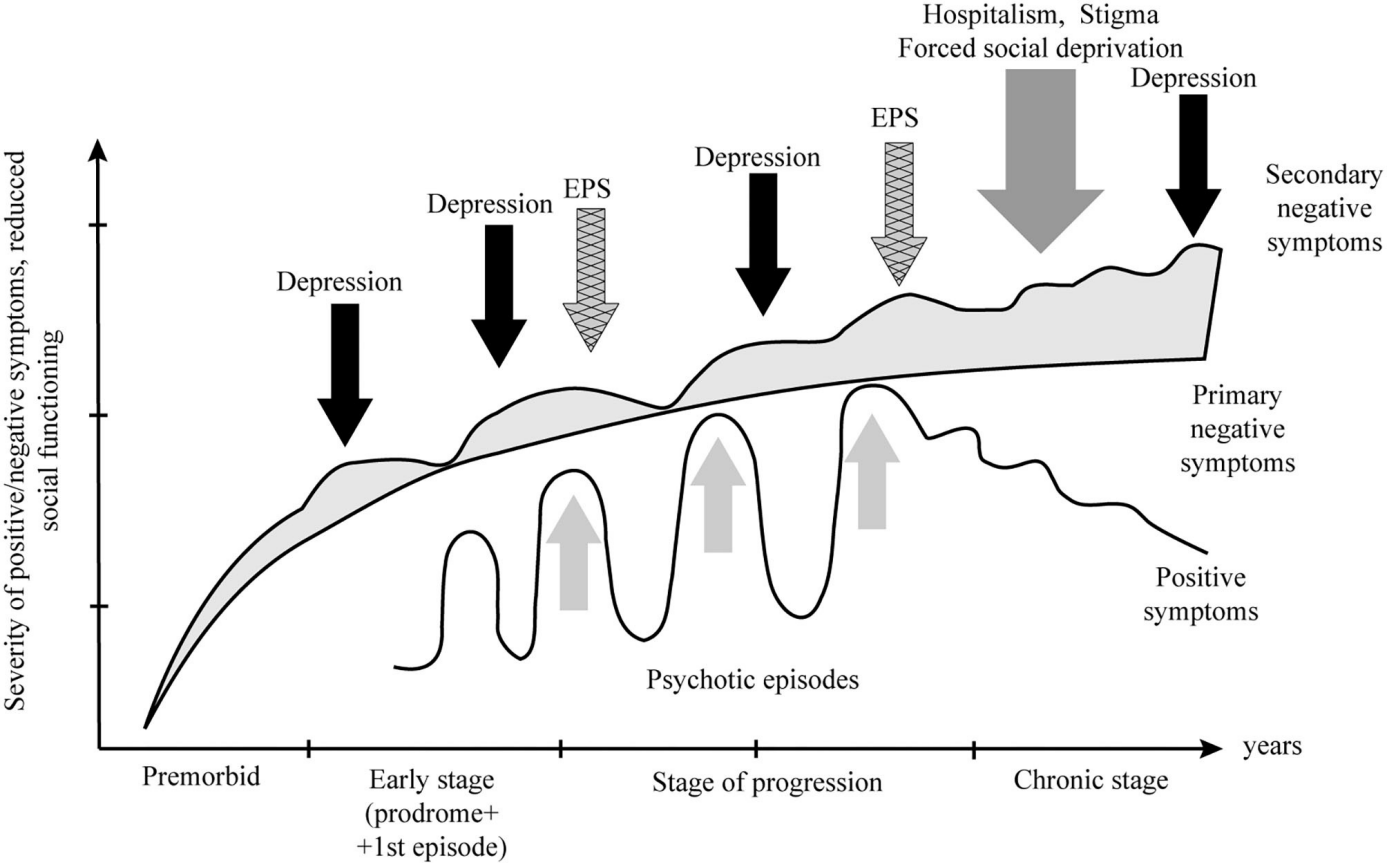
Trajectory of the development of primary and secondary negative symptoms in schizophrenia. EPS, extrapyramidal symptom.

Another critical parameter for differentiating primary and secondary negative symptoms is the dynamics or trajectory of negative symptom development (Figure 3). The matter is that primary or deficit negative symptoms are fairly stable, hardly change in the course of the disease, and are often detected at the premorbid stage. Their structure and, specifically, the ratio of motivational-volitional and emotional domains, as a rule, remain unchanged. The severity of negative symptoms either increases or remains unchanged and directly correlates with the level of the patient's functional and social disability. At the same time, the intensity of secondary negative symptoms constantly undulates depending on the patient's condition, for instance, due to the development of depression, EPS, or psychotic symptoms. In this case, secondary negative symptoms overlap with primary negative symptoms, and this can create a false clinical impression of worsening deficit symptoms and disease progression, which frequently leads to the choice of incorrect therapeutic strategy and, namely, the intensification of antipsychotic dopamine-blocking treatment.

## Conclusion

Negative symptoms lead to a significant burden and deterioration of the quality of life in patients with schizophrenia. In everyday clinical practice, negative symptoms cannot be easily recognized, and this requires focused research and the use of special psychometric scales. While the mechanisms of secondary negative symptoms are related to external causes, the pathophysiological mechanisms of primary negative symptoms are unknown and the subject of intensive research. Their understanding will help to improve pharmacotherapy and, perhaps, facilitate a better understanding of the pathogenesis of schizophrenia in general.

Managing negative symptoms in schizophrenia is a major challenge for psychiatric services. It is important to differentiate between primary and secondary negative symptoms to select the correct therapeutic tactics. When secondary negative symptoms are present, it is recommended to manage their cause, primarily comorbid depressive symptoms, and extrapyramidal disorders. When dealing with primary negative symptoms, therapeutic options should include changes in treatment regimen by adding some atypical antipsychotics with a proven “anti-negative symptom effect” (partial D2/D3 agonists may have selective benefit), specific psychotherapy [cognitive behavioral therapy (CBT), cognitive rehabilitation, Metacognitive Reflection and Insight Therapy (MERIT)], exercise physical activity, transcranial magnetic stimulation, and possibly other alternative therapies (58, 85, 86). Considering promising studies in this field, it is worth highlighting a focus on primary negative symptom specificity in different stages, forms, and subpopulations of schizophrenia patients in future development and validation of novel highly sensitive psychometric scales and identification of genetic and neurochemical markers, which should aim to establish the pathogenesis of negative symptoms, and develop more effective targeted therapy for negative symptoms.

## Limitations

Due to the limited number of journal articles on this subject, the authors focused only on the historical and conceptual aspects of negative symptomatology in schizophrenia, especially on the clinical distinction between primary and secondary negative symptoms, and did not consider cognitive, genetic, or other neurobiological aspects and pharmacological and non-pharmacological therapies. The latter includes psychotherapeutic interventions targeting cognitive deficit, which is an important predictor for the worse social functioning and therapeutic response of negative symptoms (85).

## Author Contributions

SM: study design, conceptualization, methodology, and writing and reviewing. PY: data collection, drafting the article, and figures and tables. All authors critically reviewed the article content and approved the final version.

## Conflict of Interest

In the past 5 years, SM has received honoraria or consultation fees from Angelini, Gedeon Richter, Janssen, Lundbeck, Abbott, Grindex, and Servier. The remaining author declares that the research was conducted in the absence of any commercial or financial relationships that could be construed as a potential conflict of interest.

## Publisher's Note

All claims expressed in this article are solely those of the authors and do not necessarily represent those of their affiliated organizations, or those of the publisher, the editors and the reviewers. Any product that may be evaluated in this article, or claim that may be made by its manufacturer, is not guaranteed or endorsed by the publisher.

## Appendix 1

Glossary of terms to the Figure 1

*Amnestic syndrome*- prominent impairment of recent and remote memory while immediate recall is preserved, reduced ability to learn new material, and disorientation in time. Confabulation may be a marked feature, but perception and other cognitive functions, including the intellect, are usually intact.

*Decline in the level of personality*- persistent drop in activity and efficiency, in a narrowing of the circle of interests, paling of the features inherent in the individual, increased fatigue, irritable weakness, indistinctly expressed dysmnestic disorders. Further personality changes lead to its regression, and to more severe degree of various clinical manifestations like extreme explosiveness, brutality, affective lability, sudden decrease in adaptation, manipulative behavior; or complacency, stupidity, lack of insight, inability to solve simple personal tasks.

*Energy potential reduction*- decrease in mental energy potential, which expressed by a reduction in mental activity, productivity, inability to actively use the available volume of knowledge, inability to learn new information while retaining a reserve of professional and other knowledge.

*Exhaustion of mental activity*- increased mental exhaustion, signs of irritability, weakness, hyperesthesia. Mental asthenia is the mildest form of negative syndrome.

*Marasmus*- fragmentation of the personality to such an extent that the individual no longer presents a unified, predictable set of beliefs, attitudes, traits, and behavioral responses; profound dementia with loss of contact with the environment and complete disappearance of interests and desires. The food and sexual instincts are preserved, but severe physical exhaustion, trophic changes in the skin, dystrophy of the internal organs, increased bone fragility could be observed. Marasmus with a full disintegration of the personality is the most severe type of negative syndrome.

*Objectively defined personality changes* – unexpected shift in patient personality obvious to the others. In mild cases, it's a hypertrophy or accentuation of existed personal traits, in more severe ones – it's a change of temperament, the entire warehouse of the personality changes with appearance of psychasthenic, hysterical, hypochondriacal and paranoid features, that were not typical of the patient before.

*Personality disharmony* – discordance and contradictoriness in personality traits. It can appear in acquired autism (schizoidism), manifested by detachment from environment, egocentrism, reflexivity, introversion, paradoxical emotional reactions and behavior, impoverished emotionality combined with fragile feelings (“wood and glass”), loss of emotional resonance, inability to react adequately to events around, and schematic thinking detached from reality. In such cases, monotonous behavior, paradoxical pedantry, absence of flexibility, a drop in activity and passive submissiveness are noted. Sometimes an unusual combination of inactivity and passivity with remarkable achievements in any professional areas due to the originality of the patient's technical, scientific or artistic positions is observed. Signs of personality disharmony can be a constant feeling of dissatisfaction with others, irritability, excessive exhaustion, decreased productive thinking, easy and superficial judgments, egocentrism, narrowing of interests. Minor life difficulties cause patients to experience prolonged states of confusion, helplessness and hopelessness.

*Personality regression*- regress in behavior and social or labor activities. It can be manifested by the persistent drop in activity, lack of spontaneity, sharp narrowing of interests, indifference to the others, mild memory problems.

*Subjectively conscious alteration of “Ego”*- subjectively perceived by patients changes in the pattern of their personality, which manifested by mild deviations in internal attitudes, emotional reactions, assessment of current events, and attitudes toward peers. Such changes are quite often not stated and not noticed by others.

*Total dementia* – global impairments of multiple higher cortical brain functions, including memory, thinking, orientation, comprehension, calculation, learning capacity, language, and judgement, usually of a chronic or progressive nature. The cognitive dysfunction is commonly accompanied, and occasionally preceded, by deterioration in emotional control, social behavior or motivation, leveling of individual personality traits. Patients are usually euphoric, prone to frivolous, often ridiculous acts, and flat humor; their behavior is inadequate to the situation.
